# Review on the Mechanical Properties and Modification Techniques of Coral Concrete

**DOI:** 10.3390/ma19020226

**Published:** 2026-01-06

**Authors:** Hongling Yu, Ao Zhang, Gang Cheng, Jiakun Zhu

**Affiliations:** 1 Architectural Engineering Institute, The College of Post and Telecommunication of WIT, Wuhan 430070, China; yuhongling@witpt.edu.cn; 2 China Construction Yipin Investment Development Co., Ltd., Wuhan 430000, China; a_zhang@whut.edu.cn; 3 School of Civil Engineering and Architecture, Wuhan University of Technology, Wuhan 430070, China; zhujiakun@whut.edu.cn

**Keywords:** coral aggregate concrete, aggregate properties, mechanical properties, modification techniques

## Abstract

Coral aggregate concrete (CAC) serves as a critical material for sustainable development in marine engineering, effectively addressing the shortage of aggregate resources in the construction of offshore islands and reefs. In this paper, the aggregate characteristics, static and dynamic mechanical properties and modification technology of CAC are systematically reviewed. Research indicates that the coral aggregates (CAs), due to its high porosity (approximately 50%), low bulk density (900–1100 kg/m^3^), and rough, porous surface, results in relatively low static compressive strength (20–40 MPa), insufficient elastic modulus, and significant brittleness in CAC. However, its dynamic performance shows the opposite advantage. Under impact loads, the energy absorption capacity is enhanced by 32.6–140.3%, compared to ordinary concrete (OC) due to the energy dissipation mechanism of pore platic deformation. Through the modification techniques, such as aggregate pre-treatment (acid washing/coating), incorporation of auxiliary cementitious materials (silica fume increases strength by 16.4%), fibre reinforcement (carbon fibres enhance flexural strength by 33.3%), and replacement with novel cementitious materials (magnesium sulphate cement improves chloride ion binding capacity by 90.7%), the mechanical properties and durability of CAC can be significantly optimised. This paper highlights gaps in current research regarding the high strain rate (>200 s^−1^) dynamic response, multi-factor coupled durability in marine environments, and the engineering application of alkali-activated materials, providing theoretical basis for future research directions.

## 1. Introduction

Within the marine engineering sector, concrete serves as the foundational material for constructing infrastructure. Aggregates constitute its core component, accounting for 70% to 80% of its volume. The immense demand for these materials directly stems from the scale of marine construction projects. However, transporting coarse and fine aggregates from inland sources not only increases project costs but also impacts project timelines. Consequently, to address resource scarcity while safeguarding ecological conservation, Scholars propose utilising locally sourced materials. This involves processing coral reefs surrounding islands into coarse and fine aggregates for direct on-site preparation of the coral concrete. This approach not only alleviates raw material supply pressures, but also significantly shortens construction timelines and reduces costs, holding considerable value for advancing island engineering projects [1,2,3,4,5,6,7].

Therefore, to explore the progress of coral concrete mechanics and modification technology in this paper, we used the advanced search function of Web of Science and CNKI database. The main search keywords are coral aggregate, concrete, mechanics and modification, and the year is set from 1995 to 2024. This paper systematically integrates the physical and chemical properties, static and dynamic mechanical behaviour, multi-path modification technology and high-performance research results of coral aggregate. The focus was on analysing deep-seated contradictions, such as the controversy surrounding carbonation mechanisms and the lack of constitutive theories. Finally, this paper proposes cutting-edge approaches involving cross-scale performance regulation and digital twin design, providing theoretical underpinnings and technical pathways for optimising the full life-cycle performance of coral concrete in island and reef engineering.

## 2. Coral Aggregate Characteristics

**Figure 1:**
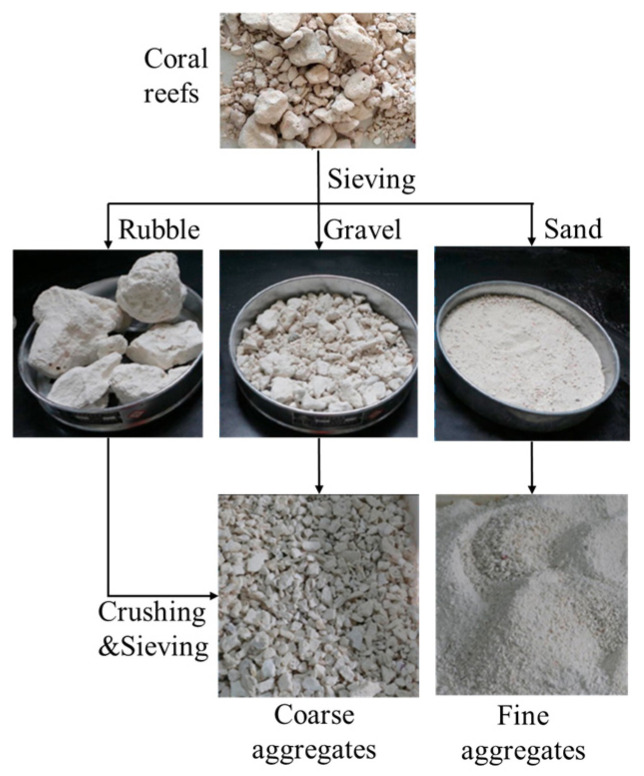
Coral debris processing [10].

**Figure 2:**
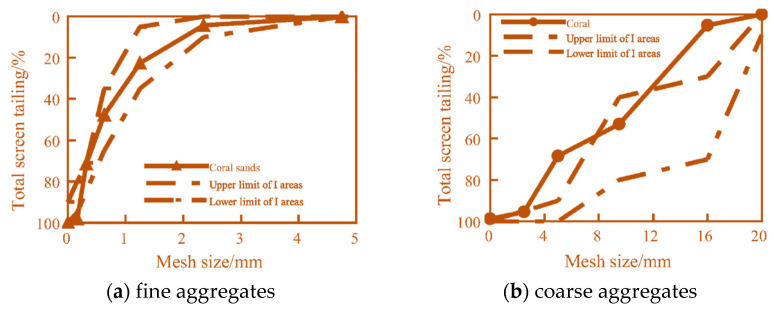
Gradation curve of coral aggregate.

Coral aggregate, or coral reef, is a calcareous rock formed through the prolonged geological processes such as sedimentation, compaction, and cementation following the death of reef-building coral polyps and their calcareous exoskeletons [8]. Its primary mineral constituents are aragonite and calcite, with calcium carbonate (CaCO_3_) constituting approximately 96% of its chemical composition. The resulting coral clasts are lightweight and porous, qualifying as natural lightweight aggregates [9]. Coral aggregates exhibit loose, porous structures containing numerous interconnected or closed pores. Compared to conventional aggregates, this high porosity results in significantly greater water absorption rates for coral aggregates. Extensive research indicates that the coral fine aggregates (CFAs) exhibit higher apparent density than coral coarse aggregates (CCAs), yet possess lower porosity and water absorption rates. As shown in Table 1, the properties of different aggregates are shown, respectively. The bulk densities of both CCAs and CFAs typically range around 900 kg/m^3^ and 1100 kg/m^3^ respectively, significantly lower than that of ordinary aggregates. Due to their high internal porosity (approximately 50%), coral aggregates exhibit markedly lower hardness and strength than conventional aggregates. As illustrated in Figure 1, reef stones typically require crushing and screening processes to meet the gradation requirements for coarse and fine aggregates in concrete. The CCAs particle sizes generally fall within the range of 5 mm to 31.5 mm. The coral aggregate gradation curve is shown in Figure 2. According to ASTM C778, CFAs are classified as fine aggregates for Zone II.

The existing classification of coral aggregate concrete (CAC) can be subdivided into three primary categories, based on the particle size and source of the aggregate replacement: The first category comprises all-coral concrete (CC), entirely manufactured from coral resources, wherein the fine aggregate consists of coral sand while the coarse aggregate comprises coral coarse aggregate [11]. The second is the seawater sea sand coral aggregate concrete (SSCAC), which utilises the sea sand as its fine aggregate, while retaining coral coarse aggregate for the coarse aggregate [12]. The third category comprises coral sand concrete (CSC), which integrates marine resources with conventional construction materials. Here, the fine aggregate originates from the coral sand, while the coarse aggregate employs traditional gravel aggregate [13].

## 3. Static Mechanical Properties of Coral Aggregate Concrete

Compressive strength is one of the most critical properties of concrete. Traditional coral aggregate concrete exhibits relatively low compressive strength, typically ranging from 20 to 40 MPa. These low-strength concretes were historically applied primarily in seawalls, road pavements, and other harbour structures and installations [3]. The strength development of CAC over time differs significantly from that of ordinary concrete (OC), with CAC demonstrating notably higher early-stage strength compared to OC [14,15]. In the existing research, most of the CAC is configured with seawater. According to ASTM D1141-2003, the content of each material per unit volume is NaCl: Na_2_SO_4_: MgCl·6H_2_O: KCl: CaCl_2_= 24.5: 4.1: 11.1: 0.7: 1.2. Research indicates that the 7-days compressive strength of seawater-mixed CAC reaches approximately 80% of its 28-days compressive strength, while the 14-days compressive strength can attain 90–95% of the 28-day value [16]. Li [17] compared the compressive strengths of CAC and OC at different curing ages under identical mix proportions. In the early stages, CAC exhibited faster strength gain than OC, though its later strength development was slower, with 28-day strength generally lower than those of ordinary concrete with the same mix design. The CAC’s 7-day compressive strength reached approximately 80% of its 28-day strength, whereas OC’s corresponding ratio was around 50–60%. At the same time, as shown in Table 2, when the mix proportions are the same, the compressive strength of CAC is still only 0.50–0.65 times that of ordinary concrete. Arumugam et al. [18] observed similar results. The difference in strength development between CAC and OC can be explained by the following mechanisms. Firstly, calcium aluminate and chloride ions in seawater react with tricalcium aluminate in cement to form Friedel salts, thereby accelerating cement hydration rates and enhancing calcium aluminate strength development [19,20]. The secondly, porous calcium aluminate absorbs water from the cement paste during the initial mixing stage, reducing the local water–cement ratio around the calcium aluminate surface. This action reduces the thickness of the water film forming on the aggregate surface, thereby decreasing porosity, mitigating interfacial transition zone (ITZ) brittleness, and limiting the enrichment of Ca(OH)_2_ crystals. Furthermore, during hydration, the water absorbed by the calcium aluminate can be released, further promoting the cement paste’s setting.

Beyond the hydration effects, the disparity in strength development may also stem from morphological differences between calcium aluminate and ordinary aggregates. Firstly, the coarser surface of CAs may induce higher friction with the hydrated cement paste. Furthermore, a portion of the cement paste may permeate into the pores of CAs. This infiltrated cement paste can enhance the properties of CAs through the formation of C-S-H gel and acicular calcium aluminate [21], and further strengthen the interfacial bonding between CAs and the cement paste. Research indicates that at 28 days, the microhardness of ITZ in CAC exceeds that of OC with identical mix proportions.

CAC performance is also significantly influenced by its raw material characteristics. Specifically, the porous structure and brittle nature of coral aggregate limit improvements in compressive strength while contributing to low elastic modulus and permeability issues. Collectively, these factors heighten CAC’s susceptibility to environmental erosion, thereby compromising its mechanical stability, practicality, and durability [22]. To enhance performance, strategies such as fibre reinforcement [23,24,25], aggregate modification [26,27], and incorporation of mineral additives (e.g., slag, fly ash, silica fume, and kaolinite) [28,29,30], and employing durable alkali-activated mortars (AAMs) or geopolymers as cement substitutes [31,32,33,34] can be used to bolster the overall performance of CAC. Existing research indicates that fibre reinforcement typically enhances the cube compressive strength of CAC by approximately 10% [25,35], while aggregate modification can increase it by around 16–20% [26,27]. Furthermore, recent years have witnessed scholarly endeavours exploring UHPC-CAC technologies. Wang et al. [33] investigated UHSC-CAC prepared based on geopolymers and the theory of dense packing. Through response surface design, CSC with 28-day compressive strengths of 135–150 MPa could be engineered.

When considering the mechanical properties of CAC, studies indicate that its splitting tensile strength, axial compressive strength, and flexural strength exhibit an approximately linear correlation with the cube compressive strength [36,37]. These mechanical properties are influenced by multiple factors, primarily including the inherent strength of the cement mortar, the intrinsic strength of the CAs, and the bond strength within the interfacial transition zone between the CAs and cement mortar [38]. Generally, the naturally low strength and brittleness of CAs, coupled with the presence of numerous micro-pores on its surface, facilitate deeper penetration of cement mortar into the CAs, thereby enhancing the bond strength. [38]. This enhances the bond strength within the interfacial transition zone, which typically exceeds the inherent strength of the CAs itself. Concurrently, the strength of cement mortar generally exceeds that of coral aggregate. Consequently, during the fracture propagation within the composite, failure typically initiates at the coral aggregate and propagates along the weakness plane, exhibiting distinct brittle behaviour [39]. It follows that the overall strength of the composite is constrained by the strength of the coral aggregate. Utilising higher-quality coral aggregate (avoiding irregularly shaped specimens such as branch-like or antler-like fragments) can significantly enhance the mechanical properties of the composite.

In contrast to its relatively low compressive strength, CAC exhibits tensile strength comparable to or exceeding that of OC [17,40]. The rough, porous surface of coral aggregates enhances mechanical interlocking between the aggregate and cement matrix at the interface. Furthermore, the micro-pumping effect [36] strengthens the concrete interface transition zone, conferring greater resistance to interfacial damage. Consequently, the tensile performance of CAC is enhanced. The incorporation of fibres further improves both the compressive and tensile properties. During micro-crack initiation and propagation, fibres act as bridges between the concrete matrix and cracks [41]. Cohesive forces between fibres and the cement matrix constrain crack expansion, stabilising it and reducing crack width and spacing [42]. Macro-cracking and aggregate failure occur when cracks propagate beyond the fibres’ capacity to maintain stable connections. Fibre presence delays the failure process and partially optimises CAC’s pore structure, thereby enhancing mechanical properties.

Under identical strength conditions, CAC exhibits a higher static elastic modulus than ordinary lightweight aggregate concrete (LAC), but lower than OC [43,44,45,46]. Li et al. [47] demonstrated that CAC exhibits a lower elastic modulus than OC, though the addition of fly ash or basalt fibres can marginally enhance concrete elastic modulus. However, the porous nature of coral aggregate accommodates greater salt crystal accumulation. Consequently, under sulphate or chloride salt wet–dry cycles, CAC’s mass and elastic modulus initially increase before decreasing [48,49]. Moreover, CAC exhibits delayed performance degradation compared to OC [49]. Additionally, Wang et al. [23,25,50,51] proposed that incorporating polypropylene fibres, carbon fibres, and sisal fibres can enhance the elastic modulus of CAC mixed with seawater by 3.7–10.6%. The static elastic modulus enhancement effect follows the following order: polypropylene fibres > carbon fibres > sisal fibres.

**Figure 3:**
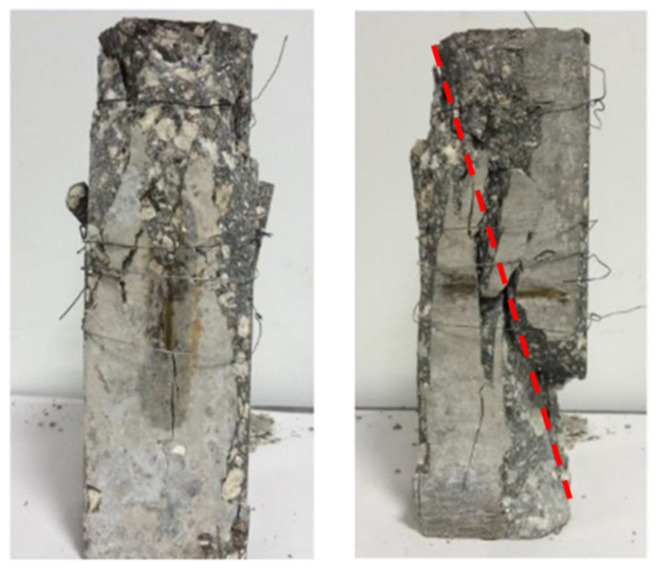
CAC uniaxial compression failure mode [55].

Unlike OC, CAC exhibits failure modes under uniaxial compression similar to high-strength concrete (HSC), albeit with significantly lower strength. During loading, localised macro-cracks initiate at coarse aggregate locations rather than from micro-cracks dispersed across the concrete surface. Upon reaching peak compressive stress, strength abruptly declines, demonstrating greater brittleness than OC at equivalent strength levels [52,53,54]. Failure cracks invariably traverse the coarse aggregate [14], typically exhibiting an inclination angle of 65° to 70° [36,55] (as illustrated in Figure 2). The failure mode of CAC is primarily attributed to two factors: firstly, the weakness of the coarse aggregate; secondly, the enhanced strength of the interfacial transition zone. Coral coarse aggregates are porous and brittle, while the presence of surface pores can enhance the strength of the transition zone. Consequently, coarse aggregates in CAC can be regarded as macroscopic defects [56]. However, due to the higher strength of the transition zone, when a crack in the cement matrix approaches coarse aggregate and propagates, it is more likely to traverse the aggregate (as illustrated in Figure 3).

**Figure 4:**
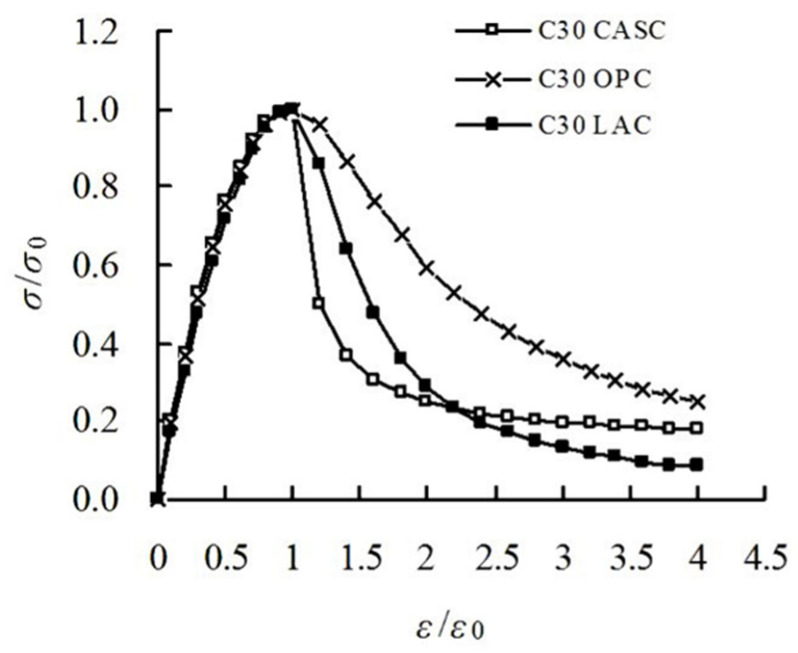
Comparison of full stress–strain curves for CAC, LAC and OC [52].

Da et al. [52] investigated the compressive behaviour of CAC at varying concrete strengths, comparing the full stress–strain curves of CAC with those of LAC and OC, as depicted in Figure 4. The study noted that the stress–strain responses of CAC, LAC, and OC exhibited similar growth trends during the initial loading phase. However, by the descending phase of the curve—where material failure commences—significant divergence emerged among the three. Specifically, after reaching the peak compressive strength, the stress–strain curve of CAC exhibited a steeper decline, revealing its greater brittleness compared to OC and LAC. This characteristics is primarily attributed to the lower density and strength of coral aggregates, rendering them more susceptible to fragmentation under compressive stress. The compressive strain behaviour of CAC, OC, and recycled aggregate concrete (RAC) was compared. Results indicate that strain in RAC and OC, prior to peak stress, primarily originates from the matrix and interface transition zone (ITZ), whereas significant strain in CAC predominantly occurs within the coarse aggregates. Crack formation within CAC was relatively infrequent until approaching the peak stress, after which cracks propagated rapidly to material failure [53]. Furthermore, following the peak stress, the stress–strain curve of CAC exhibited a markedly higher decay rate than both RAC and OC, indicating a more pronounced brittle failure mode for CAC [57].

## 4. Dynamic Mechanical Properties of Coral Aggregate Concrete

**Figure 5:**
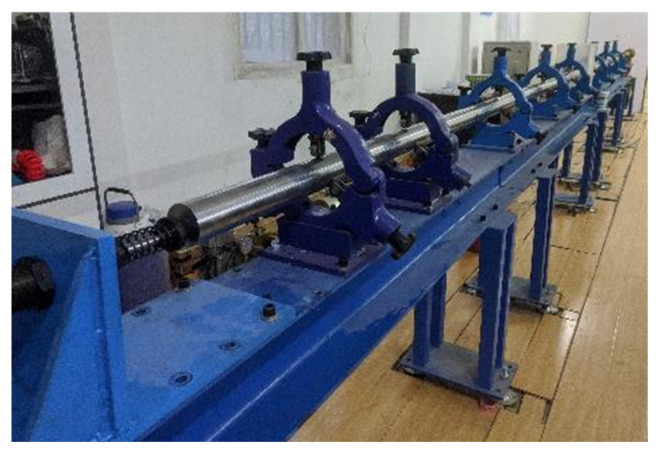
SHPB test equipment.

**Figure 6:**
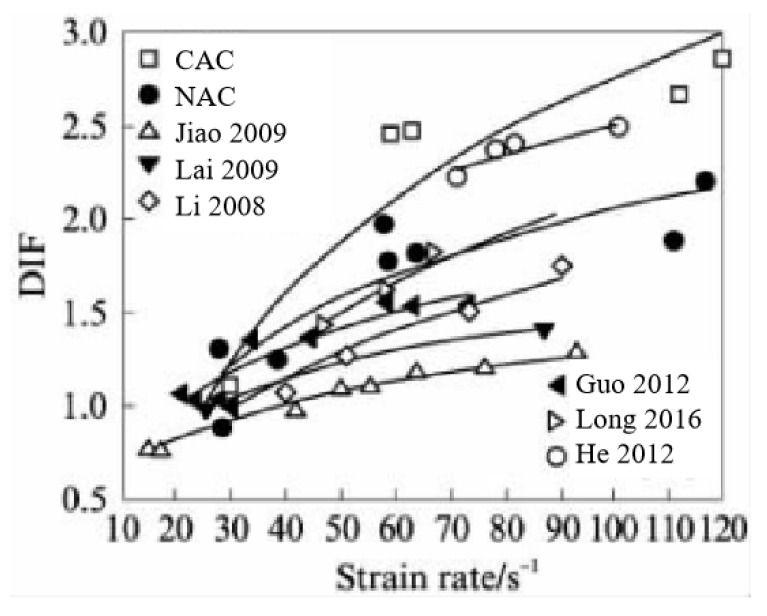
Comparison of DIF between CAC and OC with strain rate [63,64,65,66,67,68].

In marine environments, concrete structures are subjected not only to quasi-static loads but to dynamic loads, as well, such as wave impact, earthquakes, tsunami impacts, and blasting. Cai et al. [58] and Xu et al. [59] reported that, at equivalent strength grades, the dynamic compressive strength of CAC exhibits greater sensitivity to strain rate than that of OC. This aligns with the findings of Ma et al. [60], who additionally observed that under both quasi-static and dynamic loading, the fracture surface of CC penetrates directly through CAs, resembling the fracture behaviour of CC under static loading. This holds significant implications for the design and construction of seismic and blast-resistant engineering on islands and reefs. Furthermore, Yue et al. [61] indicated that the dynamic increment factor (DIF) of C50 CAC exceeds that of OC when strain rates range between 30 and 150 s^−1^. Wu et al. [62] obtained the dynamic constitutive relationship of CAC using a Hopkinson pressure bar (SHPB, as shown in Figure 5) and compared it with OC of the same strength grade. As illustrated in Figure 6, the results indicate that, at identical strain rates, CAC exhibits superior energy absorption capacity, enhanced impact resistance, and greater fragmentation [62,63,64,65,66,67,68]. This may stem from the high porosity and low strength of the CAs matrix, where numerous irregularly shaped voids within the aggregate–cement matrix induce stress concentration. These regions are prone to plastic deformation, fracture, and slip, a process that aids in dissipating and absorbing vibrational or impact energy applied to the structure. Furthermore, the free water and air within the pores undergo compression under external loading and expansion during unloading. This dynamic change provides an inherent flexible buffering mechanism for the material. During this process, a portion of the external load’s energy is converted into frictional heat energy within the air and kinetic energy within the water, subsequently dissipating gradually throughout the system. Consequently, CAC exhibits superior fatigue and impact resistance compared to OC. Fu et al. [69] reported that the strain rate effect on the dynamic compressive strength of CAC becomes more pronounced with increasing water saturation, indicating enhanced flexible buffering effects from water-filled pores. This further corroborates the aforementioned conclusions.

Wang et al. [70] incorporated polypropylene fibres into high-strength coral concrete and subjected it to free-fall impact testing. Their findings revealed that polypropylene fibre addition effectively enhanced the impact resistance of the high-strength coral concrete, increasing the number of impacts sustained and demonstrating a linear relationship between absorbed energy and fibre dosage. Concurrently, Yi et al. [71] arrived at similar conclusions. Furthermore, Wang et al. [72] observed that carbon fibre addition significantly improved the impact resistance of coral concrete while effectively reducing its brittleness. Research by Qin et al. [73] demonstrated that carbon nanotubes and basalt fibres can enhance the dynamic compressive strength and toughness of CAC, achieving maximum strength increases of 106% and 73%, respectively. The optimal content ratios for carbon nanotubes and basalt fibres were determined to be 0.04% and 0.3%. Furthermore, Liu et al. [74] observed that the dynamic compressive strength of CAC containing 1.2% organic fibres increased from 157.49 MPa to 247.99 MPa, representing a 32.6% improvement. As the organic fibre content increased, the toughness indices of coral concrete specimens grew, alongside higher fracture integrity. To enhance the energy absorption capacity and impact toughness of CAC, Ma et al. [75] reinforced it with 0.2% steel fibres. They observed that at strain rates of 42.1–137.4 s^−1^the energy absorption of CAC was 49.6–140.3% higher than that of unfibred concrete. Wang Zhenbo et al. [76] investigated the impact performance of concrete materials reinforced with either PVA fibres alone or a composite of PVA and steel fibres. They observed that fibre addition significantly mitigated the brittleness of SSCAC and enhanced its dynamic mechanical response.

Regarding the failure modes, Wu et al. [77] and Ma et al. [60] quantitatively analysed the fragmentation extent of C40 CAC under impact compression loads via SHPB testing. They found that as the strain rate increased from 30.12 s^−1^ to 143.32 s^−1^, the fractal dimension of CAC rose from 2.027 to 2.302. This indicates a heightened degree of fragmentation and self-similarity within the CAC. Wu et al. [78] further observed that larger specimen dimensions correlated with increased crack density and failure area.

Beyond experimental investigations, Ma et al. [75] employed LS-DYNA software to analyse the dynamic compressive behaviour of high-strength CAC. The simulated stress–strain curves and failure modes exhibited good agreement with experimental results. The simulations further indicated that CAC’s Poisson’s ratio exhibited no strain rate effect within the strain rate range of 3 × 10^−6^ to 1.8 × 10^2^ s^−1^. Wu et al. [78] proposed a novel three-dimensional mesoscale simulation method for numerical investigations utilising random aggregate properties, analysing the coupled effects of strain rate (10^−3^–200 s^−1^) and specimen size on the dynamic compressive strength of CAC. Subsequently, a three-dimensional regression function was derived based on the simulation results. Subsequently, Wu et al. [79] employed a three-dimensional stochastic micromodel integrating coral aggregate, cement mortar, and their interfacial transition zone to construct a three-phase stochastic micromodel of concrete. The findings demonstrated that this three-dimensional stochastic micromodel and its corresponding material parameters exhibited high reliability in predicting the mechanical properties of CAC, effectively describing its damage and failure patterns under loading. This holds significant importance for analysing the failure mechanisms of CAC under various loading conditions. Subsequently, Ma et al. [80] investigated the static and dynamic mechanical properties of CAC before and after high-temperature exposure, along with its penetration resistance and blast performance. Numerical simulations were conducted using the aforementioned three-dimensional stochastic aggregate micromodel. Results indicated that both static and dynamic mechanical properties of CAC were significantly reduced following high-temperature exposure, with simulation outcomes showing good agreement with experimental data.

## 5. Stress–Strain Relationship of Coral Aggregate Concrete

### 5.1. Uniaxial Compression Stress–Strain Curves

Numerous mathematical function forms, including polynomial, exponential, trigonometric, and rational fraction equations, have been proposed both domestically and internationally [81]. These equations are generally characterised by simplicity and practicality.

(1) $$y=2x+x^{2}(\mathrm{Hognestad})$$

(2) $$y=c_{1}x+c_{2}x^{2}+c_{3}x^{3}+c_{4}x^{4}(\mathrm{Sacnz})$$

(3) $$y=xe^{1-x}(\mathrm{Sahlin}\text{-}\mathrm{Smith}\text{-}\mathrm{young})$$

(4) $$y=6.75\left(e^{-0.812x}-e^{-1.128x}\right)(\mathrm{Umemura})$$

(5) $$y=\sin\left(\frac{\pi }{2}x\right)(\mathrm{Young})$$

(6) $$y=\sin\left[\frac{\pi }{2}\left(-0.27\left|x-1\right|+0.73x+0.27\right)\right](\mathrm{Okayama})$$

(7) $$y=\frac{\left(c_{1}+1\right)x}{c_{1}+x^{2}}(\mathrm{Tulin}\text{-}\mathrm{Gerstle})$$

(8) $$y=\frac{c_{1}x+\left(c_{2}-1\right)x^{2}}{1+\left(c_{2}-2\right)x+c_{2}x^{2}}\;(\mathrm{Sargin})$$

(9) $$y=\frac{2x}{1+x^{2}}(\mathrm{Desayi})$$

The concrete compressive stress–strain mathematical models proposed by the above scientists all adopt the unified model of the ascending and descending segments. Although this unified model is simple in form and convenient in calculation, the fitting accuracy is not high in most cases, and it is difficult to describe the geometric characteristics of the whole curve. At present, the most widely used in China is the piecewise expression equation proposed by Professor Guo Zhenhai of Tsinghua University, which can accurately describe the characteristics of concrete compressive stress–strain curve. The 2002 edition of China’s Code employs the full stress–strain curve for uniaxially compressed concrete proposed by Guo Zhenhai of Tsinghua University, expressed as follows:

(10) $$y(x)=\left\{\begin{array}{l} \alpha_{a}x+\left(3-2\alpha_{a}\right)x^{2}+\left(\alpha_{a}-2\right)x^{3}\;x\leq 1\\ \frac{x}{\alpha_{d}{\left(x-1\right)}^{2}+x}\;\;\;\;\;\;\;\;\;x>1 \end{array}\right.$$

where $x=\varepsilon /\varepsilon_{0}$, ε represents strain, $\varepsilon_{0}$ denotes peak strain, $y=\sigma /\sigma_{0}$, *σ* represents stress, $\sigma_{0}$ denotes peak stress, a is the control parameter for the ascending phase curve, and b is the control parameter for the descending phase curve.

### 5.2. Dynamic Constitutive Equations

The dynamic constitutive model of concrete and its impact resistance form a crucial foundation for the design and analysis of military protective engineering structures. Under high-velocity impact, concrete exhibits rate-dependent strengthening behaviour in its dynamic strength, with stress–strain curves displaying similarity across different strain rates. However, the transient, high-intensity nature of complex impact loads such as high-speed collisions and explosions poses significant challenges to research on concrete’s dynamic performance. Current representative concrete constitutive models employed for impact numerical simulations include the following:

(1) J-H/HJC model [82]: Developed by Holmquist et al., specifically designed for concrete target plate penetration problems. This model is extensively cited in Chinese literature, spurring numerous studies on constitutive model refinements and numerical simulations for impact penetration processes.

(2) Forrestal model [83]: Derived from the Johnson–Cook metallic strength model, it simultaneously accounts for pressure-induced nonlinear effects and temperature influences.

(3) RHT model [84]: Proposed by Riedel et al., its core lies in representing the evolution of concrete’s initial yield strength, failure strength, and residual strength through three distinct strength envelopes: the elastic limit surface, failure surface, and residual strength surface.

(4) Malvar Model [85]: An enhanced version of the original concrete/geomaterial model within LS-DYNA software(V.17.2), it now serves as the built-in concrete damage constitutive model for this software.

From a damage mechanics perspective, the aforementioned models essentially remain phenomenological empirical formulations, lacking a foundation in thorough micro-mechanical mechanism analysis. The definition of damage within these models, its evolution laws, and the mechanisms through which it influences constitutive behaviour urgently require further investigation integrated with micro-mechanism exploration. To establish an empirical dynamic constitutive model for concrete exposed to full coral seawater, considering both strain rate hardening and damage softening effects, Fan [86] proposed modifying the Ottosen nonlinear elastic constitutive model to derive dynamic constitutive equations. The proposed dynamic constitutive equations can be expressed as follows:

(11) $$\sigma =f\left(\sigma_{s},R_{rate},D\right)=(1-D)R_{rate}\sigma_{s}$$

where σ denotes dynamic compressive strength, *D* represents the dynamic damage weakening factor, $R_{rate}$ is the strain rate strengthening factor, and $\sigma_{s}$ denotes static compressive strength. The values of $R_{rate}$, *D*, and $\sigma_{s}$ can be obtained from the following formula:

(12) $$\begin{array}{l} R_{rate}=\left(a_{1}+a_{2}\bar{\dot{\varepsilon }}\right)\\ D=h(D_{0}+m\varepsilon^{n})\\ \sigma_{s}=\frac{b_{1}\frac{\varepsilon }{\varepsilon_{p}}+(b_{2}-1){\left(\frac{\varepsilon }{\varepsilon_{p}}\right)}^{2}}{1+(b_{1}-2)\frac{\varepsilon }{\varepsilon_{p}}+b_{2}{\left(\frac{\varepsilon }{\varepsilon_{p}}\right)}^{2}}f_{cs} \end{array}$$

where $\bar{\dot{\varepsilon }}$ denotes the average strain rate, $a_{1}$ and $a_{2}$ represent strain rate intensification coefficients, ε signifies the dynamic strain value, $D_{0}$ indicates the initial damage of the concrete material, *h* denotes the crack closure coefficient, *m* and *n* are material constants, $\varepsilon_{p}$ denotes the peak dynamic impact strain, $f_{cs}$ represents the quasi-static uniaxial compressive strength, $b_{1}=E_{0}/E_{1}$ (where $E_{0}$ is the initial elastic modulus of concrete and $E_{1}$ is the secant modulus at the point where concrete stress reaches the quasi-static uniaxial compressive strength), $b_{2}$ is a coefficient, and $b_{2}$ exerts little influence on the ascending segment of the dynamic stress–strain curve but significantly affects the descending segment. A higher value of $b_{2}$ results in a more gradual decline in the curve’s descending segment.

## 6. Coral Concrete Modification Technology

### 6.1. Pre-Treatment of Aggregates

The limestone produced by coral polyps forms rock through prolonged compaction; hence, the upper layers of coral reefs are slightly softer than the lower strata due to having undergone a shorter period of consolidation. Coral aggregates typically contain ions such as chloride and sulphate, resulting from prolonged immersion in seawater. Furthermore, due to natural harvesting, coral aggregates often bear attached silt and marine microorganisms on their surfaces, which can affect the strength of the composite [87].

A common method for treating coral aggregate involves immersing it in a low-concentration weak acid solution. Yao et al. [88] employed dilute hydrochloric acid to remove loose surface material from coral stone, thereby generating new reactive surfaces. The treated coral stone is then further immersed in a water glass solution, where the adhered Na_2_SiO_3_ reacts with hydrated Ca(OH)_2_ to generate additional C-S-H gel and form a denser interfacial transition zone. Research findings indicate that the CAC prepared with modified aggregates can achieve a compressive strength of 45 MPa at 28 days. Lü [34] employed acetic acid solution for CAs pre-treatment, finding that solution concentration and immersion time must be controlled to achieve suitable properties. He recommended an acetic acid concentration below 3% and immersion duration under 60 min to prevent damage to the aggregate’s skeletal structure. Concurrently, Wang et al. [27] reached similar conclusions, successfully producing high-strength geopolymer coral aggregate concrete with 28-day compressive strength exceeding 60 MPa through low-concentration acetic acid pre-treatment. Chu [89] investigated citric acid treatment of coral aggregates, finding that modified coral aggregates significantly enhanced the mechanical properties of magnesium sulphate cement-based coral concrete (CSCB). Compared to untreated coral aggregate, the 28-day-old magnesium sulphate cement-based CSCB exhibited a 20.5% increase in flexural strength and a 31.8% rise in compressive strength. Furthermore, the workability of this material was markedly improved, with an increase of 35.3%. Wang [33] employed phosphoric acid immersion treatment on coral aggregates, observing that the modified coral concrete exhibited extended initial and final setting times compared to the untreated control.

Beyond acid treatment, some researchers have utilised coating solvents for modification. Lü [34] applied geopolymer cementitious materials for pre-coating coral aggregate. Owing to its high fluidity, this effectively filled the pores within the aggregate, while the surface geopolymer layer provided additional lubrication during mixing. Guo et al. [26] utilised polyvinyl alcohol (PVA) solution to enhance CA properties, significantly reducing porosity and increasing bond strength with cement. The modified CAC exhibited 21% and 16.9% increase in compressive strength at 7 and 28 days, respectively. Liu et al. [90] introduced organic solvent-based silane coupling agents (SCAs) to enhance aggregate properties. Their research demonstrated that SCAs effectively reduced the crushing index and water absorption of coarse coral aggregates, improved adhesion between aggregates and mortar, enhanced interfacial microhardness, and increased compressive strength. Liu et al. [91] further employed composite surface modification of aggregates using blast furnace slag and sodium silicate, observing that both agents effectively reduced aggregate porosity, accelerated cement hydration, and elevated concrete compressive strength.

Beyond surface treatments, Huang [92] modified coral aggregates using various nanomaterials and vacuum mixing. The study demonstrated that nano-CaCO_3_, nano-SiO_2_, and nano-TiO_2_ all increased the apparent and bulk densities of coral aggregates while significantly reducing water absorption and porosity. Process optimisation during concrete production can also enhance the strength of coral aggregate concrete (CAC). Chen’s [93] research indicates that pre-mixing coral aggregate, river sand, and a portion of water for approximately 10 s before adding cement and continuing mixing ensures uniform cement paste distribution over aggregate surfaces. This process inhibits potential agglomeration and enhances cement hydration. Consequently, the strength of the prepared concrete increases with improved adhesion between aggregate and cement matrix. Typically, these treatment methods enhance the strength of the prepared concrete. This is achieved by removing impurities adhering to the CAs, reducing its porosity, and strengthening its bond with the cement matrix. In summary, these approaches demonstrate that appropriate pre-treatment can improve the strength of CAC. However, considering the associated time and labour costs, implementing these methods in field applications remains challenging.

### 6.2. Adding Supplementary Cementitious Materials

The use of supplementary cementitious materials (SCMs) can effectively enhance concrete’s mechanical properties and durability, among other characteristics [94,95,96,97]. The most widely applied SCMs include fly ash (FA), blast furnace slag (BFS), silica fume (SF), and metakaolin (MK), which primarily improve concrete performance through micro-filling effects and pozzolanic activity. Consequently, the properties of marine aggregate concrete (CAC) can be enhanced by increasing the proportion of SCMs.

Chen et al. [98] investigated the effect of blended mineral admixtures on the mechanical properties of marine aggregate concrete. Results indicated that marine aggregate concrete blended with metakaolin and silica fume exhibited superior mechanical performance. Zhu et al. [99] demonstrated that introducing mineral admixtures significantly improves the composition of hydration products and the submicroscopic pore structure within the cement paste, increasing concrete compressive strength by 17.7%. Sun’s [100] research demonstrated that adding 30% SF increased the 3-day and 28-day compressive strengths of CAC by 23.9% and 16.4%, respectively. Su et al. [29] found that incorporating silica fume and slag significantly reduced the capillary water absorption of CAC, with 4% silica fume or 20% slag effectively lowering its porosity. Cheng et al. [101] noted that combining SCMs with water-saturated CAs can reduce CAC porosity and optimise its microstructure, enhancing compressive strength while reducing chloride ion permeability. Results further indicate that incorporating MK yields more pronounced strengthening effects compared to FA and BFS, owing to MK’s higher pozzolanic activity [102]. Wang et al. [103] compared the effects of SF and BFS on the mechanical properties and chloride ion resistance of CC. Results indicated that CC incorporating SF exhibits stronger chloride ion binding capacity due to SF’s higher reactivity and specific surface area.

Furthermore, with the increasing application of nanomaterials, recent studies have employed these materials to enhance the performance of coral aggregate cementitious systems. Zhang [104] investigated the fresh-mix properties, printability, and mechanical performance of 3D-printed seawater coral sand mortar incorporating nano-silica (NS). Findings indicated that NS addition improved the extrusion, deformation, and constructability of the printed mortar. Guo et al. [105] investigated the dynamic mechanical properties of nano-SiO_2_-reinforced coral sand cement mortar under varying confining pressures and strain rates by compounding nano-SiO_2_ with basalt fibres. Chen [106] investigated the impact resistance of coral sand cementitious matrixes by adding carbon nanotubes. Results indicated that specimen strength and toughness reached maximum enhancement at a carbon nanotube content of 0.4%.

### 6.3. Fibre Blending

Existing research indicates that compared to ordinary concrete (OC), carbon-fibre-reinforced concrete (CAC) exhibits greater brittleness [52] and lower fracture toughness [56]. Fibres play a crucial role in enhancing concrete’s toughness, crack resistance, and impact resistance, effectively improving its mechanical properties [107,108]. Fibre types commonly employed in CAC include polypropylene fibres, basalt fibres, carbon fibres, and sisal fibres. Wang et al. [70] employed polypropylene fibres to prepare high-strength coral concrete, demonstrating that increasing the polypropylene fibre dosage markedly elevated the number of initial cracking impacts. Wei et al. [109] investigated the effects of carbon fibres and polypropylene fibres on the porosity, strength mechanism, compressive failure characteristics, and microstructure of high-strength coral concrete (HSCC). Results indicated that fibres enhanced the compressive strength of HSCC, though the improvement in axial compressive strength was modest. At a fibre dosage of 3 kg/m^3^, carbon fibre and polypropylene fibre increased the compressive strength of HSCC by 10.2% and 6.1%, respectively. Wang et al. [50] investigated the effects of adding sisal fibre, polypropylene fibre, and carbon fibre on the mechanical properties of cementitious composites (CACs). Results indicated that fibre addition enhanced the compressive strength, tensile strength, flexural strength, and elastic modulus of the composites, with the most pronounced improvement observed in flexural strength. Failure mode analysis revealed that fibre incorporation increased the ductility of the concrete. Liu et al. [110] demonstrated that the optimal carbon fibre dosage was approximately 1.5% by weight of the cementitious matrix. At this optimal dosage, the ultimate flexural strength, peak deflection, and flexural toughness of CCs across different strength grades increased by 20.8–33.3%, 116.2–42.1%, and 367–586%, respectively. Niu et al. [111] and Wang et al. [112] investigated the effects of basalt fibres on CC strength and durability. At certain dosages, basalt fibres effectively enhance concrete compressive strength and splitting tensile strength. Furthermore, SEM analysis revealed that basalt fibres form a tight bond with CAC, thereby restricting micro-crack initiation. However, the compressive strength enhancement of these fibres in coral concrete remains limited. Consequently, researchers have explored the use of steel fibres [33] or blended steel-fibre-with-other-fibre systems [113]. Yet, owing to steel fibres’ poor durability in marine environments, the resulting steel-fibre-reinforced coral concrete exhibits similarly inadequate durability characteristics when exposed to seawater.

### 6.4. Adopting Novel Cementitious Materials as Substitutes for Portland Cement

Given the instability of ordinary Portland cement hydration products in marine environments, selecting superior binding agents represents another novel approach. Owing to the characteristics of coral aggregates—lightweight, porous, rough-surfaced, and high-salinity—there is an urgent need to develop and adopt novel cementitious materials with enhanced strength and durability for marine applications to improve the durability and mechanical properties of coral concrete [114].

(1) Basic magnesium sulphate cement (BMSC)

Basic magnesium sulphate cement (BMSC) represents a modification of cementitious materials based on the magnesium sulphate cement system. Wu et al. [115] altered the hydration reaction process of MgO·MgSO_4_·H_2_O by introducing specific admixtures to generate a novel insoluble basic magnesium sulphate phase whisker product (5Mg(OH)_2_·MgSO_4_·7H_2_O). The whiskers form a layered structure with MgO_6_ octahedra as the framework, filled with sulphate ions, hydroxide ions, and water molecules. This hydration product exhibits low density, high strength, and high elastic modulus.

In practical engineering projects, low-strength alkaline magnesium sulphate cement concrete has been validated for use as non-load-bearing wall material, while high-strength variants are suitable for structural components [116]. Current research on coral concrete produced using BMSC primarily originates from Professor Yu Hongfa’s team at Nanjing University of Aeronautics and Astronautics and Professor Wang Aiguo’s team at Anhui University of Architecture. Their findings indicate that BMSC-based all-coral seawater concrete demonstrates superior tensile and flexural properties compared to equivalent all-coral seawater concrete made with ordinary Portland cement. Although the elastic modulus of BMSC-based all-coral seawater concrete is comparable to that of ordinary Portland cement-based all-coral seawater concrete, under identical mix proportions, BMSC concrete achieves respective increases of 16%, 75%, 41%, and 40%, respectively, compared to ordinary Portland cement concrete [14,37]. Further investigations revealed that when the strain rates ranged from 56 to 137 s^−1^, the dynamic enhancement factor of CASC incorporating BMSC and sisal fibres increased by 1.19 to 1.29 times compared to CASC using ordinary Portland cement. This demonstrates that under dynamic loading conditions, the combination of BMSC and sisal fibres significantly enhances the overall performance of coral concrete [117]. Guo [118] further investigated the post-high-temperature static and dynamic mechanical properties of magnesium sulphate cement-based coral concrete. Chu [89] demonstrated that magnesium sulphate cement-based all-coral concrete exhibits strong durability.

The BMSC possesses excellent reinforcement protection properties. Due to its favourable molecular structure, it resists Cl^−^ penetration, the primary ion causing steel corrosion. Comparing MCPC and CPC coral concretes, the apparent chloride diffusion coefficient Da of coral concrete decreases sharply with prolonged exposure time, with MSC exhibiting a faster decline rate than Portland cement. At the same depth, the free chloride ion concentration Cf (MCPC) < Cf (CPC) [119]. At 90 days of age and identical mix proportions, MCPC exhibited a 90.7% higher chloride ion binding capacity than CPC [120], demonstrating magnesium sulphate cement’s effective chloride ion sequestration.

(2) Alkali-activated geopolymer cement

Alkali-activated geopolymer cement represents a novel polymeric inorganic cementitious material. They form through the activation of solid alumina- and silica-containing precursor materials under alkaline conditions. This formation involves the cleavage of Si-(Al)-O bonds, initial polymerisation into oligomers, and subsequent polymerisation into a three-dimensional Si-O-Al network. Extensive research indicates that geopolymers are considered amorphous equivalents of zeolites [121,122], exhibiting high bond strength, low porosity [123], early strength, and high ultimate strength. At normal temperature conditions, a geopolymer was prepared by mixing a solution containing 8% Na_2_O·nSiO_2_ with kaolinite. This polymer achieved a strength of 115.6 MPa at 3 days, which further increased to 131.9 MPa at 7 days [124]. Within alkali-activated systems, three types are distinguished based on calcium oxide (CaO) content: calcium-free systems (CaO < 1.0%), low-calcium systems (CaO < 10%), and high-calcium systems (CaO > 10%). Those are represented by metakaolin (MK), fly ash (FA), and slag (S), respectively. When comparing alkali-activated slag concrete with alkali-activated fly ash concrete, the former typically exhibits higher early-stage strength. However, from a long-term performance perspective, the strength of alkali-activated slag concrete often diminishes over time [125]. For MK/S and FA/S binary mixtures, after activation treatment, the strength of the mixture increases significantly with rising slag content. The C-S-H formed by alkali-activated slag and the aluminosilicate network formed by MK and FA can coexist [126,127], with increased slag content markedly enhancing strength [128,129].

When comparing ordinary Portland cement to alkali-activated cement, the latter exhibits superior stability and lower permeability. This material demonstrates strong resistance to corrosive ions present in the environment, such as chloride ions, sulphates, and magnesium salts. Particularly, fly ash-based geopolymers, owing to their low calcium content and structural compactness, exhibit lower chloride ion penetration depths than silicate concrete. The penetration depth of chloride ions exhibits a linear decrease trend with increasing compressive strength [130]. Studies indicate that alkali-activated fly ash geopolymers show no significant deterioration after two years of immersion in NaCl solution [131]. Within low-calcium systems, geopolymers typically demonstrate outstanding sulphate resistance. Research indicates that partially substituting fly ash with kaolinite effectively mitigates strength loss in fly ash-based geopolymers [132]. When exposed to a mixed solution containing 5% Na_2_SO_4_ and 5% MgSO_4_, silicate cement specimens suffered severe compressive strength degradation, whereas geopolymer samples exhibited minimal changes in mass and strength [133]. With extended reaction times, increased Si/Al ratios enhance compressive strength. Specimens immersed in 5% Na_2_SO_4_ solution for 90–180 days exhibit slowed strength gain due to Si leaching [134]. Notably, Na_2_SO_4_ may function as an activator within the activation system under certain conditions, explaining why some experiments observed no strength loss or even slight increases in specimens. As geopolymers lack Ca(OH)_2_ and AFm, they avoid the cracking mechanisms associated with gypsum and aluminate hydrates formed in Portland cement.

Wang [33] prepared coral concrete using phosphate-modified coral aggregates and geopolymer cementitious materials. The study demonstrated that geopolymer-based coral concrete exhibits outstanding resistance to chloride salt erosion. Lü et al. [34] successfully produced high-strength coral concrete with 28-day compressive strength exceeding 60 MPa using a geopolymer based on the FA/BFS binary system. Peng et al. [135] developed inorganic polymer coral sand concrete (IPCSC) and tested its fundamental mechanical properties. Results indicated that at equivalent strength grades, IPCSC exhibited a 62% increase in splitting tensile strength, a higher tensile–compressive strength ratio, and a slight improvement in static elastic modulus. Subsequently, the effects of inorganic polymer cement replacement rate, water–binder ratio, and superplasticiser dosage on mix workability and compressive strength of coral sand concrete were investigated. Results indicated the water–binder ratio exerted the most significant influence on concrete workability [32]. Zhang et al. [136] compared the mechanical properties of alkali-activated mortars (AAMs) incorporating river sand, sea sand, and coral sand. Coral sand AAM specimens exhibited slightly higher compressive strength than river sand and sea sand AAM specimens, indicating that alkali-activated materials can fully compensate for strength losses associated with coral sand application. Zhang et al. [137] investigated the compressive behaviour of alkali-activated calcium carbonate (AACAC) and cementitious calcium carbonate (CAC) by incorporating alkali-activated materials (AAMs) as substitutes for ordinary Portland cement (OPC). Results indicate that AACAC and CAC exhibit similar failure modes under uniaxial compression, characterised by complete crushing of the coral aggregate. Furthermore, AAM incorporation improves the interfacial microstructure between coral aggregate and binder matrix [136]. This enhanced behaviour retards crack propagation, thereby increasing concrete’s splitting tensile strength and elastic modulus [138]. Compared to CAC, the slope of the stress–strain curve during the ascending phase of AACAC was not significantly different at equivalent concrete strengths. However, AACAC exhibited a slower rate of stress decline during the descending phase, indicating superior ductility.

Alkali-activated materials demonstrate immense application potential in marine engineering due to their unique properties. Furthermore, geopolymers have shown exceptional performance in shoreline stabilisation treatments and as materials for restoration works [139]. Integrating the characteristics of alkali-activated materials with marine concrete, this approach is regarded as a promising pathway to enhance the performance of coral concrete. Nevertheless, before scaling up these materials for large-scale application, a series of durability-related challenges—including volume stability, crack formation, and carbonation—must be overcome.

## 7. Conclusions and Outlook

### 7.1. Conclusions

(1) Aggregate–Matrix Interface Dominates Failure Behaviour

The high open porosity (48–52%) of CAs combined with its surface microtopography creates a ‘mechanical interlocking effect’, increasing the microhardness of the interface transition zone (ITZ) by 18–25% compared to ordinary concrete (OC). However, CaCO_3_ cleavage planes act as stress concentration points, causing cracks to propagate preferentially through the aggregate under uniaxial compression (fracture angle 65–70°), exhibiting quasi-brittle fracture characteristics.

Under dynamic loading, the hydraulic cushioning effect of pores (DIF increases 1.29-fold at water saturation >80%) combined with plastic slip along irregular pore walls achieves an energy absorption density of 4.2 kJ/m^3^ (140.3% higher than OC), revealing the dialectical unity of static brittleness and dynamic toughness.

(2) Reconstructing Cementitious Systems to Optimise Performance Boundaries

Crystalline whiskers formed by basic magnesium sulphate cement (BMSC) create a three-dimensional interlocking structure, reducing Ca(OH)_2_ enrichment in the interfacial transition zone (ITZ) by 67% and achieving chloride ion binding capacity of 0.38 wt% (90.7% higher than ordinary Portland cement, OPC), overcoming the ion diffusion limitations of traditional Portland cement.

Alkali-activated geopolymers (FA/BFS system) undergo [SiO_4_]^−^-[AlO_4_]^5−^ network polymerisation reactions, reducing porosity to the 12.3 nm level and achieving a 60 MPa-level surge in 28-day compressive strength. However, high-calcium slag systems face long-term phase transformation-induced strength decline risks (180-day loss rate >15%).

(3) Fibre-Nano Synergistic Toughening Mechanism

Carbon nanotubes (0.4 wt%) and basalt fibres (0.3 vol%) form a multi-level reinforcement network: nanotubes inhibit micro-crack initiation (critical crack width <50 μm), while fibres bridge macro-cracks (toughness index I_10_ increased by 586%), and their synergistic action achieves a peak dynamic compressive strength of 247.99 MPa (a 57.4% improvement over the control group).

### 7.2. Outlook

To advance the application of coral concrete to higher levels and meet the developmental needs of building a maritime power, the following prospects are proposed for coral aggregate concrete research:

(1) Establish a multi-field coupled damage model for extreme environments

Urgent development is required for a coupled constitutive equation integrating ‘chloride ion erosion-wet/dry cycling-high strain rate loading’ to quantify the chemo-mechanical synergistic effects of seawater ions (SO_4_^2−^/Mg^2+^) at dynamic crack tips. Key breakthroughs must address the influence of CAs phase transformation kinetics (aragonite → calcite) on energy dissipation mechanisms at strain rates exceeding 200 s^−1^.

(2) Development of Micro/Nano-Scale Interface Engineering

Grafting nano-SiO_2_ particles with silane coupling agents (SCAs) to enhance CAs-C-S-H gel chemical bonding; designing microcapsule-controlled release mineralisers (e.g., urease) to induce calcium carbonate self-deposition at cracks (biomineralisation repair rate ≥0.2 μm/d); developing ITZ directional functionalisation techniques.

(3) Intelligent Servicing Regulation of Novel Cementitious Materials

Topologically disordered structure regulation of geopolymers: Optimising [Si]/[Al] ratios (1.5–2.0) and alkali metal types (K^+^ > Na^+^) via molecular dynamics simulations to suppress volume expansion caused by N-A-S-H gel conversion to zeolite phases.

Optimisation of crystallisation kinetics in BMSC: Employing rare earth doping (La^3+^) to inhibit excessive MgO hydration, resolving the conflict between early strength development and long-term volume stability.

(4) Digital Twins and Cross-Scale Design

Establishing a morphological gene bank for coral aggregates: Mapping pore fractal dimension (D = 2.027–2.302) against compressive strength via CT scanning, combined with machine learning to predict regional coral resource suitability.

Developing discrete element models for multiphase composites: Integrating random aggregate distribution (RAS), fibre orientation tensors, and interstitial transition zone (ITZ) thickness gradients to achieve programmable performance design for UHPCAC (150 MPa grade).

## Figures and Tables

**Table 1:** Physical properties of aggregates.

Material	Bulk Density/ (kg · m^−3^)	Apparent Density/ (kg · m^−3^)	1 h Water Absorption/%	Porosity/%	Chloride Ion Content/wt%
**CCAs**	900–978	1690–1800	45.8–50	13–16.2	0.14
**FCAs**	1125–1200	2280–2530	41.3–52.0	7.1–18.5	0.07
**River sand**	1469	2563	0.45	/	/
**Conventional gravel**	1463	2756	0.86	0.60	/

**Table 2:** Comparison of compressive strength between CAC and OC [17].

No.	Materials/(kg)			Sand Ratio/(%)	Water–Cement Ratio	Compressive Strength/(MPa)	
	Cement	Aggregate	Water			OC	CAC
1	400	790	150	36	2.67	47.7	26.2
**2**	400	750	175	40	2.29	40.0	25.3
**3**	400	690	200	44	2.00	34.1	22.4
**4**	450	790	200	40	2.25	39.2	24.6
**5**	450	750	150	44	3.00	54.5	30.1
**6**	450	690	175	36	2.57	45.7	28.6
**7**	500	790	175	44	2.86	51.6	32.6
**8**	500	750	200	36	2.50	44.3	28.8
**9**	500	690	150	40	3.33	61.2	30.5

## Data Availability

No new data were created or analyzed in this study. Data sharing is not applicable to this article.

## References

[1] Wang Y. (1988). Feasibility of Coral Concrete Application in Harbour Works. Water Transp. Eng..

[2] Chen Z., Chen T., Qu J. (1991). Feasibility Study on the Application of Coral Reef Sand Concrete. Mar. Eng..

[3] Liang Y., Lu B., Huang S. (1995). Tropical Marine Environment and Marine Concrete. Mar. Technol..

[4] Wang L., Zhao Y., Lü H. (2012). Fundamental Properties and Research Prospects of Coral Aggregate Concrete. Concrete.

[5] Wang Y., Ou Z., Liu J., Chen Y., Nie Y. (2018). Research Progress on Concrete Production Using Sea Sand and Coral. Concrete.

[6] Zhang Y. (2020). Application of Coral Aggregate Concrete in Island Construction. Jiangxi Build. Mater..

[7] Lyu B., Wang A., Zhang Z., Liu K., Xu H., Shi L., Sun D. (2019). Coral Aggregate Concrete: Numerical Description of Physical, Chemical and Morphological Properties of Coral Aggregate. Cem. Concr. Comp..

[8] Cai X.G., Zhao Q., Chen H.S. (2021). Research Progress on Coral Concrete. Acta Silic. Sin..

[9] Yang Z., Qin Y., Xue C., Xiao X., Ding J., Chen G. (2024). Experimental Study on the Generalised Dynamic Modulus of Saturated Marine Coral Sand Subjected to Different Cyclic Loading Patterns. Ocean Eng..

[10] Wei J., Chen Z., Liu J., Liang J., Shi C. (2023). Review on the Characteristics and Multi-Factor Model between Pore Structure and Compressive Strength of Coral Aggregate. Constr. Build. Mater..

[11] Sun Z., Zhang L., Niu D., Wen B., Luo D. (2020). Time-Varying Model for Predicting the Resistivity of Coral Aggregate Concrete. Constr. Build. Mater..

[12] Dong Z., Wu G., Zhu H., Zhao X.-L., Wei Y., Qian H. (2020). Flexural Behaviour of Seawater Sea-Sand Coral Concrete–UHPC Composite Beams Reinforced with BFRP Bars. Constr. Build. Mater..

[13] Cheng S., Shui Z., Sun T., Yu R., Zhang G. (2018). Durability and Microstructure of Coral Sand Concrete Incorporating Supplementary Cementitious Materials. Constr. Build. Mater..

[14] Yuan Y. (2015). Mix Design and Fundamental Properties of All-Coral Seawater Concrete. Master’s Thesis.

[15] Chen Z., Zhou J., Chen Y., Yao R. (2020). Experimental Study on Mechanical Properties of Seawater Concrete with Coral Coarse Aggregate. J. Appl. Mech..

[16] Zhao Y., Han C., Zhang S., Ge R. (2011). Experimental Study on Compressive Ageing Strength of Coral Concrete Cured in Seawater. Concrete.

[17] Li L. (2012). Investigation into the Fundamental Properties of Coral Aggregate Concrete. Ph.D. Thesis.

[18] Arumugam R., Ramamurthy K. (1996). Study of Compressive Strength Characteristics of Coral Aggregate Concrete. Mag. Concr. Res..

[19] Huang L., Nima F., Shi C. (2018). The Role of Seawater in Interaction of Slag and Silica Fume with Cement in Low Water-to-Binder Ratio Pastes at the Early Age of Hydration. Constr. Build. Mater..

[20] Adel Y., Usama E., Prannoy S., Antonio N. (2018). Fresh and Hardened Properties of Seawater-Mixed Concrete. Constr. Build. Mater..

[21] Cheng S. (2016). Design, Preparation and Performance Study of Reef Sand Aggregate Concrete. Master’s Thesis.

[22] Liu B., Zhou J., Wen X., Guo J., Zhang X., Deng Z., Wang H. (2019). Experimental Investigation on the Impact Resistance of Carbon Fibres Reinforced Coral Concrete. Materials.

[23] Wang L., Wang G.X., Deng X.L. (2014). Experimental Study on the Mechanical Properties of Carbon Fibre-Reinforced Coral Concrete at Different Dosages. China Rural Water Hydropower.

[24] Wu Q., Shi W.H., Zou Y., Xu Y., You Y., Wang G., Wang S., Li X. (2020). Effect of Different Chopped Fibres on the Mechanical Properties of Coral Concrete. Concr. Cem. Prod..

[25] Wang L., Xiong Z., Liu C., Li Q. (2014). Study on the Mechanical Properties of Coral Concrete with Polypropylene Fibre Admixture. Concrete.

[26] Guo T., Ou Z., Tang J., Liu J. (2017). Experimental Study on PVA-Modified Cementitious Coral Aggregate. Contemp. Chem. Ind..

[27] Wang A., Lyu B., Zhu Y., Liu K., Guo L., Sun D. (2021). A Gentle Acid-Wash and Pre-Coating Treatment of Coral Aggregate to Manufacture High-Strength Geopolymer Concrete. Constr. Build. Mater..

[28] Su L., Niu D.T., Luo Y., Huang D.G., Luo D.M. (2021). Chloride Diffusion and Water Absorption Behaviour in Fly Ash-Coral Aggregate Concrete. Chin. J. Build. Mater..

[29] Su L., Niu D., Huang D., Zhang Y., Qiao H. (2023). Capillary Water Absorption Performance Enhancement and Prediction Model for Coral Aggregate Concrete. Mater. Rep..

[30] Deng Z., Liu B., Sheng J., Wen X., Zhou J. (2020). Research progress on coral concrete. Bull. Chin. Soc. Ceram..

[31] Xu W., Yang S., Sun H., Xu C. (2019). Study on the Mechanical Properties of Seawater Coral Aggregate Concrete with Alkali-Activated Mineral Powder. J. Xi’an Univ. Archit. Technol. (Nat. Sci. Ed.).

[32] Peng Z., Peng S., Li D. (2018). Experimental Study on Workability of Cement-Inorganic Polymer Coral Reef Sand Concrete. Concrete.

[33] Wang X. (2021). Design, Preparation and Performance Study of Geopolymer-Based High-Performance All-Coral Concrete. Master’s Thesis.

[34] Lü B. (2019). Preparation and Performance Study of Geopolymer-Based Coral Concrete. Master’s Thesis.

[35] Cui Y., Zheng Y., Rao L. (2020). Experimental Study on Mechanical Properties of Basalt Fibre Coral Concrete. Concrete.

[36] Mi R., Yu H., Ma H., Da B., Yuan Y., Zhang X., Zhu H., Dou X. (2016). Experimental Study on Mechanical Properties of Seawater Concrete with All-Coral Aggregate. Ocean Eng..

[37] Wu Z., Yu H., Ma H., Zhang Y., Mei Q., Da B., Tan Y., Hua S. (2018). Experimental Study on Mechanical Properties of Novel Coral Seawater Concrete. Mar. Eng..

[38] Diao Y., Ma H., Yu H., Zhang L., Hua S. (2021). Strength of the Interface Zone between Coral Concrete Aggregate and Clean Grout. J. Build. Mater..

[39] Ma L.J., Luo Z.M., Duan L.Q., Li Z., Zhou C. (2021). Brittleness Evaluation of All-Coral Aggregate Concrete. J. China Univ. Min. Technol..

[40] Wu W., Wang R., Zhu C., Meng Q. (2018). The Effect of Fly Ash and Silica Fume on Mechanical Properties and Durability of Coral Aggregate Concrete. Constr. Build. Mater..

[41] Wang L., Deng X.L., Wang G.X. (2014). Experimental Study on Mechanical Properties of Carbon Fibre-Reinforced Coral Aggregate Concrete. Concrete.

[42] Banthia N., Gupta R. (2006). Influence of Polypropylene Fibre Geometry on Plastic Shrinkage Cracking in Concrete. Cem. Concr. Res..

[43] Chen H.M., Liu Y.T., Guan J.W. (2021). Research progress on the durability of coral concrete reinforced with BFRP fibres. Bull. Chin. Soc. Ceram..

[44] Yao R. (2021). Experimental Investigation and Analysis of Mechanical Properties of Marine Concrete Members Reinforced with GFRP Bars and Corrosion-Resistant Steel Bars. Doctoral dissertation.

[45] Deng Z., Zhong X., Liu B., Wen K. (2022). Study on Flexural Behaviour of Coral Concrete Beams Reinforced with CFRP Bars. Structures.

[46] Gong W., Yu H., Ma H., Da B. (2019). Mix Design and Evaluation Method for Coral-Based Seawater Concrete. Mater. Rep..

[47] Li Y.T., Zhou L., Zhang Y., Cui J.W., Shao J. (2013). Study on Long-Term Performance of Concrete Based on Seawater, Sea Sand and Coral Sand. Adv. Mater. Res..

[48] Su C.Y. (2017). Study on Durability of Concrete Mixed with Seawater and Coral Reef Sand. Master’s Thesis.

[49] You W.G. (2020). Long-Term Performance of CFRP-Reinforced Coral Concrete Under Marine Tidal Action. Master’s Thesis.

[50] Wang L., Liu C., Xiong Z. (2014). Experimental Study on Mechanical Properties of Sisal Fibre-Reinforced Coral Concrete. J. Henan Polytech. Univ. (Nat. Sci. Ed.).

[51] Wang L., Yi J., Deng X., Li J. (2016). Study on Mechanical Properties and Failure Mechanisms of Fibre-Reinforced Coral Concrete. J. Henan Polytech. Univ. (Nat. Sci. Ed.).

[52] Da B., Yu H., Ma H., Tan Y., Mi R., Dou X. (2016). Experimental Investigation of Whole Stress-Strain Curves of Coral Concrete. Constr. Build. Mater..

[53] Huang Y., He X., Sun H., Sun Y., Wang Q. (2018). Effects of Coral, Recycled and Natural Coarse Aggregates on the Mechanical Properties of Concrete. Constr. Build. Mater..

[54] Wang G., Wei Y., Miao K., Zheng K., Dong F. (2022). Axial Compressive Behaviour of Seawater Sea-Sand Coral Aggregate Concrete-Filled Circular FRP-Steel Composite Tube Columns. Constr. Build. Mater..

[55] Zhang Y. (2017). Research on Static and Dynamic Mechanical Properties of Coral-Based Seawater Concrete. Master’s Thesis.

[56] Yang S., Zhang X., Yu M., Yao J. (2019). An Analytical Approach to Predict Fracture Parameters of Coral Aggregate Concrete Immersed in Seawater. Ocean Eng..

[57] Huang Y., Li X., Lu Y., Wang H., Wang Q., Sun H., Li D. (2019). Effect of Mix Component on the Mechanical Properties of Coral Concrete under Axial Compression. Constr. Build. Mater..

[58] Cai Y., Ren H., Long Z., Guo R., Du K., Chen S., Zheng Z. (2022). Comparison Study on the Impact Compression Mechanical Properties of Coral Aggregate Concrete and Ordinary Portland Concrete. Structures.

[59] Xu J., Tang Y., Chen Y., Chen Z. (2022). Experimental Study on Dynamic Mechanical Properties of Seawater Marine Aggregate Concrete Using Hopkinson Bar. Vib. Shock.

[60] Ma L., Li Z., Liu J., Duan L., Wu J. (2019). Mechanical Properties of Coral Concrete Subjected to Uniaxial Dynamic Compression. Constr. Build. Mater..

[61] Yue C., Yu H., Ma H., Mei Q., Liu T. (2021). Experimental investigation and numerical simulation of the impact compression behaviour of all-coral-water concrete. J. Build. Mater..

[62] Wu W.J., Wang R., Zhu C.Q., Meng Q.S., Liu H.F. (2019). Experimental Study on Dynamic Compressive Properties of Coral Aggregate Concrete. Chin. J. Build. Mater..

[63] Jiao C., Sun W., Huan S., Jiang G. (2009). Behavior of steel fiber-reinforced high-strength concrete at medium strain rate. Front. Archit. Civ. Eng..

[64] Lai J., Sun W. (2009). Dynamic behaviour and visco-elastic damage model of ultra-high performance cementitious composite. Cem. Concr. Res..

[65] Li W., Xu J., Shen L., Li Q. (2008). Dynamic mechanical properties of basalt fiber reinforced concrete using a split Hopkinson pressure bar. Acta Mater. Compos. Sin..

[66] Guo Y., Liu F., Chen G., Zeng G., Liu F. (2012). Experimental investigation on impact resistance of rubberized concrete. J. Build. Mater..

[67] Long G., Li N., Xue Y., Xie Y. (2016). Mechanical properties of self-compacting concrete incorporating rubber particles under impact load. J. Chin. Ceram. Soc..

[68] He Y.-M., Huo J., Chen B.-S., Huang Z.-Y. (2012). Impact tests on dynamical behavior of concrete at elevated temperatures. Eng. Mech..

[69] Fu Q., Zhang Z., Zhao X., Hong M., Guo B., Yuan Q., Niu D. (2021). Water Saturation Effect on the Dynamic Mechanical Behaviour and Scaling Law Effect on the Dynamic Strength of Coral Aggregate Concrete. Cem. Concr. Comp..

[70] Wang L., Fan L.-B., He Y.-L., Zhang J.-W. (2023). Experimental Study on Impact Resistance of High-Strength Coral Concrete. Concrete.

[71] Yi J., Liu C., Wang L. (2019). Experimental study on impact resistance of polypropylene fibre-reinforced coral concrete. Sci. Technol. Eng..

[72] Wang L., Gu W., Wang R., Liu C. (2019). Experimental Study on Impact Resistance of Carbon Fibre-Reinforced Coral Concrete. Bull. Chin. Soc. Ceram..

[73] Qin Y., Xu D., Zhang S., Fan X. (2022). Dynamic behaviour of carbon nanotubes and basalt fibre reinforced coral sand cement mortar at high strain rates. Constr. Build. Mater..

[74] Liu J., Zhang S., Zhou T., Yin Q., Xie W. (2023). Influence of Polyvinyl Alcohol and Ultra-High Molecular Weight Polyethylene Fibres on the Dynamic Mechanical Properties of Full-Coral Concrete and Numerical Simulation. J. Compos. Mater..

[75] Ma H., Yue C., Yu H., Mei Q., Chen L., Zhang J., Zhang Y., Jiang X. (2020). Experimental Study and Numerical Simulation of Impact Compression Mechanical Properties of High Strength Coral Aggregate Seawater Concrete. Int. J. Impact Eng..

[76] Wang Z., Li P., Han Y., Hao R.S., Ding X.P. (2022). Experimental Study and Simulation of Impact Compression Properties of Fibre-Reinforced Coral Aggregate Concrete. Acta Chim. Sin..

[77] Wu J.W., Ma L.J., Kong X., Luo Z., Duan L. (2020). Dynamic Characteristics of Coral Aggregate Concrete Under Impact Loading. J. Build. Mater..

[78] Wu Z., Zhang J., Yu H., Ma H., Chen L., Dong W., Huan Y., Zhang Y. (2020). Coupling Effect of Strain Rate and Specimen Size on the Compressive Properties of Coral Aggregate Concrete: A 3D Mesoscopic Study. Compos. Part B Eng..

[79] Wu Z.Y., Zhang J.H., Yu H.F., Ma H.Y., Fang Q. (2021). Simulation of Mechanical Properties of Coral Concrete Based on a Three-Dimensional Stochastic Microscopic Model. Acta Silic. Sin..

[80] Ma H.Y., Yu H.F., Guo J.B., Mei Q.Q., Yue C.J. (2019). Static and dynamic mechanical properties and numerical simulation of all-coral seawater concrete. J. Build. Mater..

[81] Guo Z.H., Zhang X.Q. (1981). Full stress-strain curve of concrete under repeated loading. Metall. Constr..

[82] Timothy J.H., Gordon R.J. (2011). A Computational Constitutive Model for Glass Subjected to Large Strains, High Strain Rates and High Pressures. J. Appl. Mech..

[83] Forrestal M., Luk V., Watts H. (1988). Penetration of Reinforced Concrete with Ogive-Nose Penetrators. Int. J. Solids Struct..

[84] Riedel W. (2000). Concrete Under Dynamic Loads: Meso- and Macro-Mechanical Models and Their Parameters.

[85] Javier Malvar L., Crawford J., Wesevich J., Don S. (1997). A Plasticity Concrete Material Model for DYNA3D. Int. J. Impact Eng..

[86] Fan F., Ye X., Xu J., Li W., Chen Y. (2010). Dynamic Constitutive Relations of Basalt Fibre-Reinforced Concrete under Impact Loading. Vib. Shock.

[87] Chai Y., Niu Y., Li W., Lü H. (2021). Research progress on modification techniques for coral aggregate concrete. Mater. Rep..

[88] Yao Y., Wang Z., Wang L., Wu H., Zhang P., Yang H. (2016). A Method for Preparing Coarse Aggregate from Coral Reef Stones and Its Concrete Application.

[89] Chu Y. (2021). Preparation and Performance Study of Magnesium Sulfate Cement-Based All-Coral Concrete. Master’s Thesis.

[90] Liu J., Ju B., Yin Q., Xie W., Xiao H., Dong S., Yang W. (2021). Properties of Concrete Prepared with Silane Coupling Agent-Impregnated Coral Aggregate and Coral Concrete. Materials.

[91] Liu J., Ju B., Xie W., Zhou T., Xiao H., Dong S., Yang W. (2021). Evaluation of the Effects of Surface Treatment Methods on the Properties of Coral Aggregate and Concrete. Materials.

[92] Huang L. (2022). Experimental Study on Basic Mechanical Properties and Water Resistance of Modified Coral Aggregate Concrete. Master’s Thesis.

[93] Chen D. (2017). Influence of Mixing Process on Properties of Porous Aggregate Concrete. Master’s Thesis.

[94] Badogiannis E., Sfikas I., Voukia D., Trezos K., Tsivilis S. (2015). Durability of Metakaolin Self-Compacting Concrete. Constr. Build. Mater..

[95] Dadsetan S., Bai J. (2017). Mechanical and Microstructural Properties of Self-Compacting Concrete Blended with Metakaolin, Ground Granulated Blast-Furnace Slag and Fly Ash. Constr. Build. Mater..

[96] Jiang J., Lu Z., Niu Y., Li J., Zhang Y. (2016). Investigation of the Properties of High-Porosity Cement Foams Based on Ternary Portland Cement–Metakaolin–Silica Fume Blends. Constr. Build. Mater..

[97] Zhang W., Hama Y., Seung H. (2015). Drying Shrinkage and Microstructure Characteristics of Mortar Incorporating Ground Granulated Blast Furnace Slag and Shrinkage Reducing Admixture. Constr. Build. Mater..

[98] Chen Z., Yao R., Zhou J., Chen Y. (2023). Experimental Study on Mechanical Properties of Marine Aggregate Concrete. Concrete.

[99] Zhu S., Shui Z., Yu R., Cheng S., Wang X. (2017). Effects of Multi-Mineral Admixtures on the Properties of Coral Sand Concrete. Bull. Chin. Soc. Ceram..

[100] Sun B. (2014). Experimental Study on Silica Fume Enhancement of Mechanical Properties in Coral Concrete. Cryog. Build. Technol..

[101] Cheng S., Shui Z., Yu R., Sun T., Zhang X. (2017). Multiple Influences of Internal Curing and Supplementary Cementitious Materials on the Shrinkage and Microstructure Development of Reef Aggregate Concrete. Constr. Build. Mater..

[102] Cheng S., Shui Z., Sun T., Yu R., Zhang G., Ding S. (2017). Effects of Fly Ash, Blast Furnace Slag and Metakaolin on Mechanical Properties and Durability of Coral Sand Concrete. Appl. Clay Sci..

[103] Wang Y., Zhang S., Niu D., Su L., Luo D. (2019). Effects of Silica Fume and Blast Furnace Slag on the Mechanical Properties and Chloride Ion Distribution of Coral Aggregate Concrete. Constr. Build. Mater..

[104] Zhang G. (2023). Study on the Properties of Cementitious Materials for Underwater 3D Printing. Master’s Thesis.

[105] Guo R., Xu X., Ren H., You Q., Sun J., Li J., Liu B. (2023). Dynamic Mechanical Properties of Nano-SiO_2_ Reinforced Coral Sand Cement Mortar under Active Confining Pressure. Acta Silic. Sin..

[106] Chen W. (2023). Experimental Study on Mechanical Properties and Self-Healing of Cementitious Matrix with South China Sea Coral Sand. Master’s Thesis.

[107] Yoo D., Banthia N. (2016). Mechanical Properties of Ultra-High-Performance Fibre-Reinforced Concrete: A Review. Cem. Concr. Comp..

[108] Navid R., Zhang M. (2020). Fibre-Reinforced Geopolymer Composites: A Review. Cem. Concr. Comp..

[109] Wei B., Wei C., Yi J. (2023). Experimental Study on Mechanical Properties of Fibre-Reinforced High-Strength Coral Concrete. J. Anhui Univ. Archit..

[110] Liu B., Guo J., Wen X., Zhou J., Deng Z. (2020). Study on Flexural Behaviour of Carbon Fibre Reinforced Coral Concrete Using Digital Image Correlation. Constr. Build. Mater..

[111] Niu D., Su L., Luo Y., Huang D., Luo D. (2020). Experimental Study on Mechanical Properties and Durability of Basalt Fibre Reinforced Coral Aggregate Concrete. Constr. Build. Mater..

[112] Wang Y., Zhang S., Niu D., Su L., Luo D. (2020). Strength and Chloride Ion Distribution Brought by Aggregate of Basalt Fibre Reinforced Coral Aggregate Concrete. Constr. Build. Mater..

[113] Yang G., Yang S., Wang Y., Xie Y. (2023). Effect of Steel-Polypropylene Hybrid Fibres on the Mechanical Properties of Coral Aggregate Concrete. J. Xiangtan Univ. (Nat. Sci. Ed.).

[114] Wang A., Lyu B., Zhang Z., Liu K., Xu H., Sun D. (2018). The Development of Coral Concretes and Their Upgrading Technologies: A Critical Review. Constr. Build. Mater..

[115] Wu C., Yu H., Dong J., Zheng L. (2014). Effects of Material Ratio, Fly Ash, and Citric Acid on Magnesium Oxysulfate Cement. ACI Mater. J..

[116] Yang S., Ma H., Yu H., Zhang N., Yang Y., Zhu H., He L., Li S. (2016). Experimental Study on Mechanical Properties of Magnesium Oxysulphate Cement Concrete. Bull. Chin. Soc. Ceram..

[117] Yue C., Yu H., Ma H., Zhang Y., Mei Q., Da B. (2019). Experimental Study on Dynamic Impact Performance of Coral-Based Seawater Concrete. Mater. Rep..

[118] Guo J. (2021). Study on Post-High-Temperature Static and Dynamic Mechanical Properties of Magnesium Sulfate-Based Cement Coral Concrete. Master’s Thesis.

[119] Da B., Yu H., Ma H., Zhang Y., Tan Y., Mi R., Dou X. (2016). Surface Free Chloride Ion Concentration and Apparent Chloride Diffusion Coefficient of Coral Concrete in Marine Environment. J. Southeast Univ. (Nat. Sci. Ed.).

[120] Da B., Yu H., Ma H., Tan Y., Mi R., Dou X. (2016). Chloride Diffusion Study of Coral Concrete in a Marine Environment. Constr. Build. Mater..

[121] Ye H., Radlińska A. (2016). Fly Ash-Slag Interaction during Alkaline Activation: Influence of Activators on Phase Assemblage and Microstructure Formation. Constr. Build. Mater..

[122] Ma X., Zhang Z., Wang A. (2016). The Transition of Fly Ash-Based Geopolymer Gels into Ordered Structures and the Effect on the Compressive Strength. Constr. Build. Mater..

[123] Singh B., Ishwarya G., Gupta M., Bhattacharyya S.K. (2015). Geopolymer Concrete: A Review of Some Recent Developments. Constr. Build. Mater..

[124] Wang A., Sun D., Hu P., Ren X. (2008). Experimental study on the preparation of geopolymer concrete using alkali-activated kaolinite. J. Hefei Univ. Technol. (Nat. Sci. Ed.).

[125] Wardhono A., Gunasekara C., Law D., Setunge S. (2017). Comparison of Long-Term Performance between Alkali-Activated Slag and Fly Ash Geopolymer Concretes. Constr. Build. Mater..

[126] Buchwald A., Hilbig H., Kaps C. (2007). Alkali-Activated Metakaolin-Slag Blends—Performance and Structure in Dependence of Their Composition. J. Mater. Sci..

[127] Yip C.K., Lukey G.C., Deventer J.S.J. (2005). The Coexistence of Geopolymeric Gel and Calcium Silicate Hydrate at the Early Stage of Alkaline Activation. Cem. Concr. Res..

[128] Zhang Y., Sun W., Chen Q., Chen L. (2007). Synthesis and Heavy Metal Immobilisation Behaviours of Slag-Based Geopolymer. J. Hazard. Mater..

[129] Pradip N., Prabir K. (2014). Effect of GGBFS on Setting, Workability and Early Strength Properties of Fly Ash Geopolymer Concrete Cured in Ambient Condition. Constr. Build. Mater..

[130] Faiz U.A. (2014). Effects of Alkali Solutions on Corrosion Durability of Geopolymer Concrete. Adv. Concr. Constr..

[131] Fernandez-Jimenez A., García-Lodeiro I., Palomo A. (2007). Durability of Alkali-Activated Fly Ash Cementitious Materials. J. Mater. Sci..

[132] Duan P., Yan C., Zhou W. (2016). Influence of Partial Replacement of Fly Ash by Metakaolin on Mechanical Properties and Microstructure of Fly Ash Geopolymer Paste Exposed to Sulfate Attack. Ceram. Int..

[133] Bakharev T. (2005). Durability of Geopolymer Materials in Sodium and Magnesium Sulphate Solutions. Cem. Concr. Res..

[134] Džunuzović N., Komljenović M., Nikolić V., Ivanović T. (2017). External Sulphate Attack on Alkali-Activated Fly Ash-Blast Furnace Slag Composite. Constr. Build. Mater..

[135] Peng Z.Q., Peng S., Li D., Fan J. (2016). Experimental Study on Basic Mechanical Properties of Inorganic Polymer-Coral Sand Concrete. J. Wuhan Univ. Technol..

[136] Zhang B., Zhu H., Shah K.W., Dong Z., Wu J. (2020). Performance Evaluation and Microstructure Characterisation of Seawater and Coral/Sea Sand Alkali-Activated Mortars. Constr. Build. Mater..

[137] Zhang B., Zhu H., Li F., Dong Z., Zhang P. (2021). Compressive Stress-Strain Behaviour of Seawater Coral Aggregate Concrete Incorporating Eco-Efficient Alkali-Activated Slag Materials. Constr. Build. Mater..

[138] Zhang B., Zhu H., Wang Q., Shah K.W., Wang W. (2022). Design and Properties of Seawater Coral Aggregate Alkali-Activated Concrete. J. Sustain. Cem.-Based Compos..

[139] Huseien G., Mirza J., Ismail M., Ghoshal S.K., Hussein A. (2017). Geopolymer Mortars as Sustainable Repair Material: A Comprehensive Review. Renew. Sustain. Energy Rev..

